# Combination of biochar amendment and phytoremediation for hydrocarbon removal in petroleum-contaminated soil

**DOI:** 10.1007/s11356-016-7236-6

**Published:** 2016-08-04

**Authors:** Tao Han, Zhipeng Zhao, Mark Bartlam, Yingying Wang

**Affiliations:** 1 Key Laboratory of Pollution Processes and Environmental Criteria (Ministry of Education), Tianjin Key Laboratory of Environmental Remediation and Pollution Control, College of Environmental Science and Engineering, Nankai University, Tianjin, 300071 China; 2 College of Life Sciences, Nankai University, Tianjin, 300071 China

**Keywords:** Petroleum hydrocarbons, Ryegrass, Biochar, Phytoremediation

## Abstract

Remediation of soils contaminated with petroleum is a challenging task. Four different bioremediation strategies, including natural attenuation, biochar amendment, phytoremediation with ryegrass, and a combination of biochar and ryegrass, were investigated with greenhouse pot experiments over a 90-day period. The results showed that planting ryegrass in soil can significantly improve the removal rate of total petroleum hydrocarbons (TPHs) and the number of microorganisms. Within TPHs, the removal rate of total n-alkanes (45.83 %) was higher than that of polycyclic aromatic hydrocarbons (30.34 %). The amendment of biochar did not result in significant improvement of TPH removal. In contrast, it showed a clear negative impact on the growth of ryegrass and the removal of TPHs by ryegrass. The removal rate of TPHs was significantly lower after the amendment of biochar. The results indicated that planting ryegrass is an effective remediation strategy, while the amendment of biochar may not be suitable for the phytoremediation of soil contaminated with petroleum hydrocarbons.

## Introduction

The rapid development of the global economy has led to considerable environmental pollution by a wide range of persistent organic and inorganic pollutants (Gaskin and Bentham 2010; Zhang et al. 2010). Petroleum products are widely used in modern society and have become one of the most important environmental pollutants (Wang et al. 2010, 2012; Zhang et al. 2010). Petroleum is a mix of different compounds, consisting mainly of saturated hydrocarbons, aromatic hydrocarbons, resins, and asphaltenes (Liu et al. 2014). Petroleum is reported to cause environmental risks in the soil ecological system (Wang et al. 2012), such as inhibition of plant growth, damage to soil structure, destruction of groundwater quality, and so on (Cai et al. 2010). Moreover, the hazardous chemicals in petroleum also pose serious threats to human health (Anyika et al. 2015).

Considerable efforts have been made for the remediation of petroleum-contaminated sites. Phytoremediation is one of the most favorable remediation techniques since it is both cost-effective and environmentally friendly (Gaskin et al. 2010; Al-Mansoory et al. 2015). Plants can have a number of effects, including degradation, transformation, assimilation, metabolism, and detoxification of hazardous pollutants from soils and aquatic and atmospheric sites (Cai et al. 2010). Several plant species, such as ryegrass, have been successfully applied to the phytoremediation of soil contaminated with organic and inorganic pollutants (Khan et al. 2013; Mimmo et al. 2015; Lu et al. 2015). The question of how to improve the efficiency and optimize the conditions of phytoremediation is one of the major concerns. It was reported that both the physicochemical properties and microbial activities of soil had a strong impact on the effectiveness of phytoremediation (Guo et al. 2014).

In recent years, the use of biochar as a soil amendment has been the subject of increasing attention (Tang et al. 2013). Biochar is formed by burning biomass under hypoxia and low temperature and is a low-density charred material (Bastos et al. 2014; Mukherjee et al. 2014; Tang et al. 2013). It was reported that biochar could change the soil physicochemical properties (Brennan et al. 2014). For example, it could increase the soil pH (Beesley and Marmiroli 2011; Mukherjee et al. 2014; Schmidt et al. 2014), strengthen the water retaining capacity of soil (Evangelou et al. 2014; Yao et al. 2012), raise the soil fertility (Mia et al. 2014; Steinbeiss et al. 2009), reduce the leaching of soluble macronutrients (Lucchini et al. 2014; Quilliam et al. 2013a), and heighten carbon sequestration (Bastos et al. 2014; Méndez et al. 2012). These lead to potentially beneficial effects on crop productivity, plant establishment, and growth; mitigating climate change by sequestrating C from atmosphere into soil; and improving moisture, nutrient retention, and microbial activity (Brennan et al. 2014). Meanwhile, biochar has also been used as a promising material in environmental remediation applications (Qin et al. 2013; Ahmad et al. 2014; Garcia-Delgado et al. 2015). For instance, biochar has been applied as a novel carbonaceous material to adsorb metals in soil and water (Beesley et al. 2010; Ahmad et al. 2014). It was reported that biochar could reduce the toxicity and mobility of a lot of toxic metals (Gomez-Eyles et al. 2011; Oleszczuk et al. 2012). Due to its high surface area and microporosity, biochar has also been proven to be efficient in adsorbing organic contaminants in water (Lou et al. 2011; Ahmad et al. 2014). Compared to water remediation, relatively limited studies are available on the application of biochar in remediation of soil contaminated with organic pollutants (Ahmad et al. 2014). Most of the studies to date have focused on the use of biochar to bind or stimulate the microbial degradation of organic pollutants (Qin et al. 2013; Xin et al. 2014). For instance, biochar amendment was shown to promote the microbial degradation of petroleum-contaminated soil (Qin et al. 2013). However, little is currently known about the effects of biochar on the phytoremediation of petroleum-contaminated soil.

The aims of the current study were (1) to assess the potential of wheat straw-derived biochar amendments, ryegrass plant, and the combination of the two strategies on the removal of petroleum hydrocarbons and (2) to elucidate the effects of biochar on the phytoremediation of petroleum-contaminated soil by ryegrass and on the number of microorganisms in soil contaminated with petroleum hydrocarbons.

## Material and methods

### Soil preparation

Soil samples were taken from an un-contaminated farming area located in the Xiqing District of Tianjin, China. The soil was air-dried and passed through a 2-mm sieve to ensure the soil homogeneity. The crude oil was evenly sprayed in the soil, with a concentration of 10,000 mg/kg (1.0 %, W_oil_/W_dry soil_). The soil was then blended and aired for 2 weeks before usage. The content of total petroleum hydrocarbons (TPHs) (day 0) was 7719 ± 113 mg/kg.

### Production and characterization of biochar

The wheat straws were air-dried and milled or grounded to pass a 20-mesh sieve. Biochar was produced at 450 °C under anaerobic conditions for 1 h using a slow pyrolysis process (Novak et al. 2009). Biochar was passed through a 100-mesh sieve before usage.

The pore structure of biochar was measured by nitrogen gas adsorption analysis at 77 K using ASAP 2020 Surface Area Analyzer (Brewer et al. 2009; Novak et al. 2009). Ash was measured at 600 °C for 6 h. Biochar was dissolved in deionized water (1 % *w*/*v*) and shaken for 24 h at 200 rpm before the pH was measured (Novak et al. 2009). Contents of C, H, N, and S elements were determined using the elemental analyzer (Euro, EA3000) (Brewer et al. 2009; Novak et al. 2009).

### Remediation treatments

The ryegrass seeds were purchased from Suqian, Jiangsu, China. The pot culture experiments were carried out in the greenhouse. There were four treatments: control (designated as C treatment); amendment of biochar (1 %) only (designated as B treatment); planted ryegrass only (designated as P treatment); and planted ryegrass together with biochar amendment (1 %) (designated as PB treatment). Each treatment had three replicates. The ryegrass seeds were germinated for 1 week at 25 °C with 70 % moisture content. Ten seedlings were then planted into the plastic pots (Φ 20 cm × 20 cm) containing 1500 g petroleum-contaminated soil. Fertilizer (251.2 mg/pot N, 157.0 mg/pot P_2_O_5_, and 188.4 mg/pot K_2_O) was applied at the beginning of the experiment. The experiments were carried out in a greenhouse with natural sunlight and a light/dark cycle of approximately 16/8 h for 90 days. Samples were taken at day 0 and day 90. The greenhouse temperature was kept at 18–25 °C, and soil moisture was maintained at 60 % of the field water-holding capacity (WHC) by daily watering.

### Total petroleum hydrocarbon analysis

The TPHs were determined as described before (Cai et al. 2010). A soil sample (100 g) was taken from each pot. Samples were air-dried at room temperature, passed through a 100-mesh sieve, and stored at 4 °C for further analysis. Five grams of soil samples was dissolved in 20 ml dichloromethane in a 40-ml glass centrifuge tube. The dichloromethane/soil suspension was agitated with a glass stirring rod for 1 min followed by extraction for 15 min using the ultrasonic method. During the extraction, the water bath was kept below 35 °C by adding cold water. The suspension was then centrifuged for 10 min at 4000 rpm. The supernatant was then transferred into an Erlenmeyer flask, which was dried to a constant weight in an oven at 105 °C in advance. The pellet was re-suspended in 20 ml dichloromethane, and the above procedures were repeated three times. All the supernatants were combined and then completely evaporated at room temperature in a fume hood. The amount of the TPHs was calculated gravimetrically. The removal rate of TPHs was determined using the following equation:

$$ \mathrm{Removal}\ \mathrm{rate}\ \left(\%\right)=\frac{TPH_0-{TPH}_{90}}{TPH_0}\times 100 $$

where TPH_0_ is the total petroleum hydrocarbons on day 0 and TPH_90_ is the total petroleum hydrocarbons after 90 days of incubation.

### Determination of alkanes and aromatic hydrocarbons

The components of TPHs, including alkanes and aromatic hydrocarbons, were fractionated by silica gel and neutral alumina column chromatography followed by gravimetric analysis. A glass column (Φ 10 mm × 300 mm) was filled with 120 mm activated silica gel (pre-baked at 120 °C for 4 h), 60 mm activated neutral alumina (pre-baked at 500 °C for 4 h), and 10 mm anhydrous sodium sulfate (pre-baked at 500 °C for 4 h). The TPHs were dissolved in a small amount of n-hexane and loaded onto the silica gel and neutral alumina column, which was pre-eluted with n-hexane. The saturated hydrocarbons were eluted with 20 ml n-hexane, followed by a 70-ml n-hexane/dichloromethane (1:1) mixture to obtain the aromatic hydrocarbons. All elutes were completely evaporated at room temperature in a fume hood.

The n-alkanes and 16 priority polycyclic aromatic hydrocarbons (PAHs) dissolved in n-hexane were filtered through a 0.22-μm nylon membrane filter and performed on a 7890 Agilent gas chromatograph coupled to a model 5975 mass selective detector (MSD; SIM mode). Hydrocarbon Window Defining Standard and PAH Solution Mix were purchased from AccuStandard, Inc. (New Haven, CT, USA). The n-alkanes were separated with a He carrier gas (1.5 ml/min) on a 60-m DB-5ms column, 0.250 mm internal diameter and 0.25 μm film thickness. The following column oven program was used for n-alkane measurements: 40 °C for 2 min, then ramped at 3 °C/min to 300 °C for 55 min. The PAHs were separated with a He carrier gas (1.0 ml/min) on a 30-m HP-5ms column, 0.250 mm internal diameter and 0.25 μm film thickness. The following column oven program was used for PAH measurements: 70 °C for 1 min, ramped at 10 °C/min to 260 °C for 4 min, and then 5 °C/min to 300 °C and held for 4 min. An external standard method was used to calculate the amount of n-alkanes and PAHs.

### Ryegrass biomass and pigment analysis

Ryegrass was harvested after the 90-day incubation. Plant samples were carefully grubbed from a pot with a handheld trowel and washed with tap water, followed by thorough rinsing with distilled water. Plants were air-dried at room temperature in the interior until the constant weight was reached. Shoot height, root length, and weight of plant were measured.

The fresh leaves were picked, cut up, and blended to measure the pigment content. Ten milliliters of extraction mixture (absolute ethyl alcohol/acetone/water 45:45:10) was added to the leaves and kept in the dark for a week at 4 °C. Chlorophyll and carotenoid contents were measured spectrophotometrically at 663, 645, and 470 nm and calculated as previously reported (Arnon 1949).

### Flow cytometry analysis

One gram of fresh soil samples was transferred into a 10-ml centrifuge tube followed by 5 ml of sterile distilled water as solvent. The mixture was vortexed for 1 min, followed by ultrasonication for 30 s (Ramsay 1984). The soil suspension was then centrifuged for 10 min at 3000 rpm. The supernatant was stained with 10 µl/ml^−1^ SYBR Green I (100× in DMSO) for 15 min at room temperature in the dark. Sample was diluted with sterile distilled water prior to flow cytometry (FCM) analysis.

A Partec CyFlow Space flow cytometer (Partec GmbH, Münster, Germany) was used to measure the number of microbial cells. A blue solid-state laser at 50 mW in FCM emits at a fixed wavelength of 488 nm. SYBR Green I was triggered on the green fluorescence at 520 ± 20 nm. Data were collected on two-parameter dot plots of green fluorescence as logarithmic signals. These measurements were made the specific instrumental gain settings—FL1 652, SSC 280, speed 3 (Ma et al. 2013). In order to calculate the moisture content, fresh soil was placed in an oven at 105 °C for 24 h, and the dry soil was weighed. The number of cells was adjusted to a dry soil weight basis.

### Statistical analysis

Data and analysis of variance procedure (one-way ANOVA) for all treatments were conducted by the Microsoft Excel software and SPSS 18.0. The values were represented as mean ± standard deviation (S.D.). The differences were analyzed at the 0.05 level.

## Results

### Characterization of biochar

Detailed information regarding the characterization of biochar is shown in Table 1. Its specific surface area (6.86 m^2^/g), micropore area (0.17 m^2^/g), total pore volume (22.29 mm^3^/g), and micropore volume (0.02 mm^3^/g) were very low compared with that of activated carbons (Brewer et al. 2009; Chen et al. 2014). Ash content and pH were 42.25 % and 10.09, respectively. Content of C element (48.45 %) was greater than that of H (1.78 %), N (1.47 %), and S (0.78 %) elements.

**Table 1:** Characterization of biochar

Index	Value
Specific surface area (m^2^/g)	6.86
Micropore area (m^2^/g)	0.17
Total pore volume (mm^3^/g)	22.29
Micropore volume (mm^3^/g)	0.02
Ash (wt%)	42.25
pH	10.09
C (wt%)	48.45
H (wt%)	1.78
N (wt%)	1.47
S (wt%)	0.78

### The removal of TPHs

**Fig. 1:**
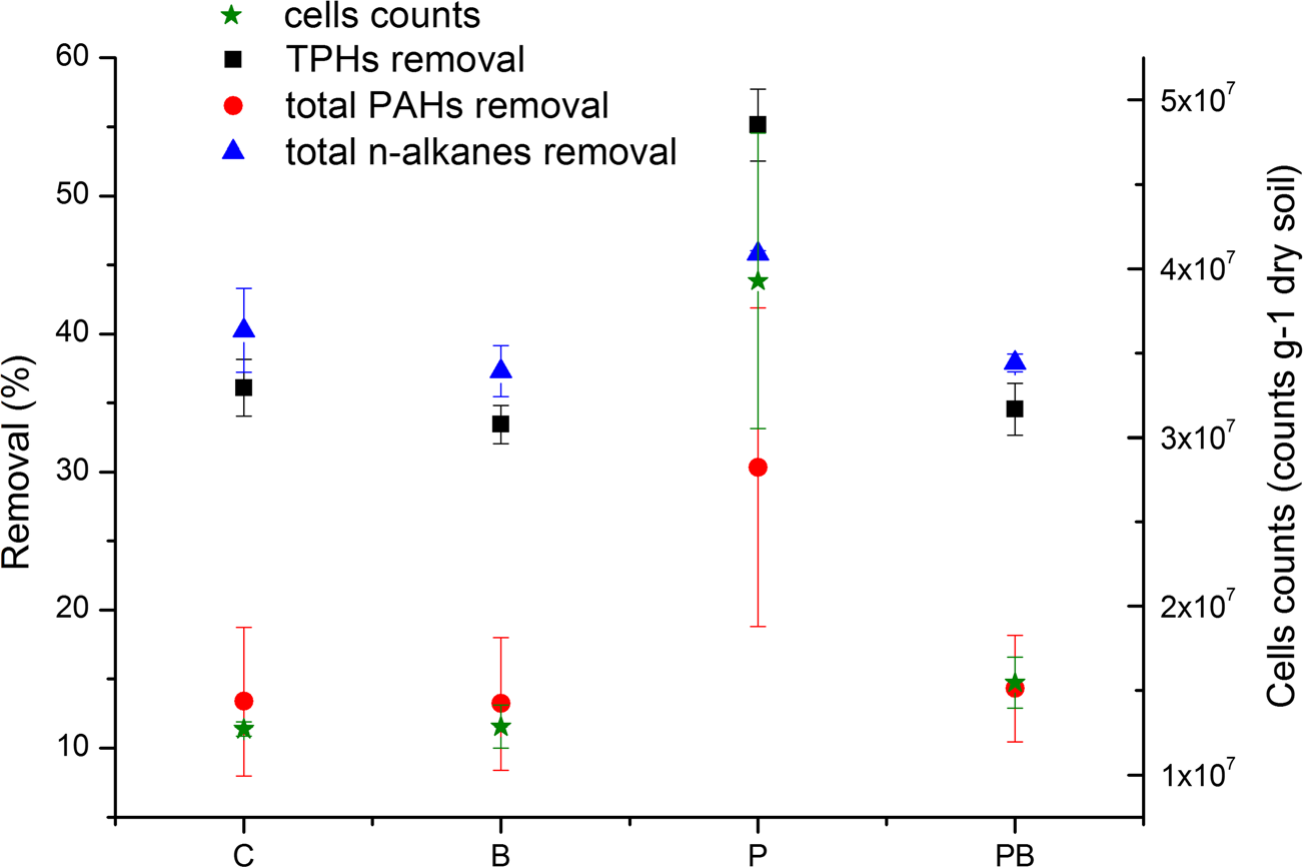
TPHs, n-alkanes, and PAH removal and microbial concentration in contaminated soil (10 g kg^−1^) under four different treatments after incubation for 90 days. *Error bars* represent the standard deviation from measurements in triplicate

The removal rate of TPHs using various treatments showed significant differences (*p* < 0.01) (Fig. 1). The removal of TPHs in soil contaminated with petroleum hydrocarbons was higher during the growth of ryegrass. The removal rate of TPHs in treatment P (55.13 %) was significantly higher (*p* < 0.01) than in the other treatments. After 90 days of incubation, the removal rate of TPHs under treatment P was 1.53, 1.65, and 1.60 times than that in treatments C, B, and PB, respectively. However, the removal of TPHs in soil contaminated with petroleum hydrocarbons was restrained by the biochar. There were no significant differences among the treatments B, PB, and C (*p* > 0.05). The removal rate in treatment B (33.45 %) was slightly lower than that in treatment C (36.08 %) and treatment PB (34.54 %).

### Growth and pigment analysis of ryegrass

**Fig. 2:**
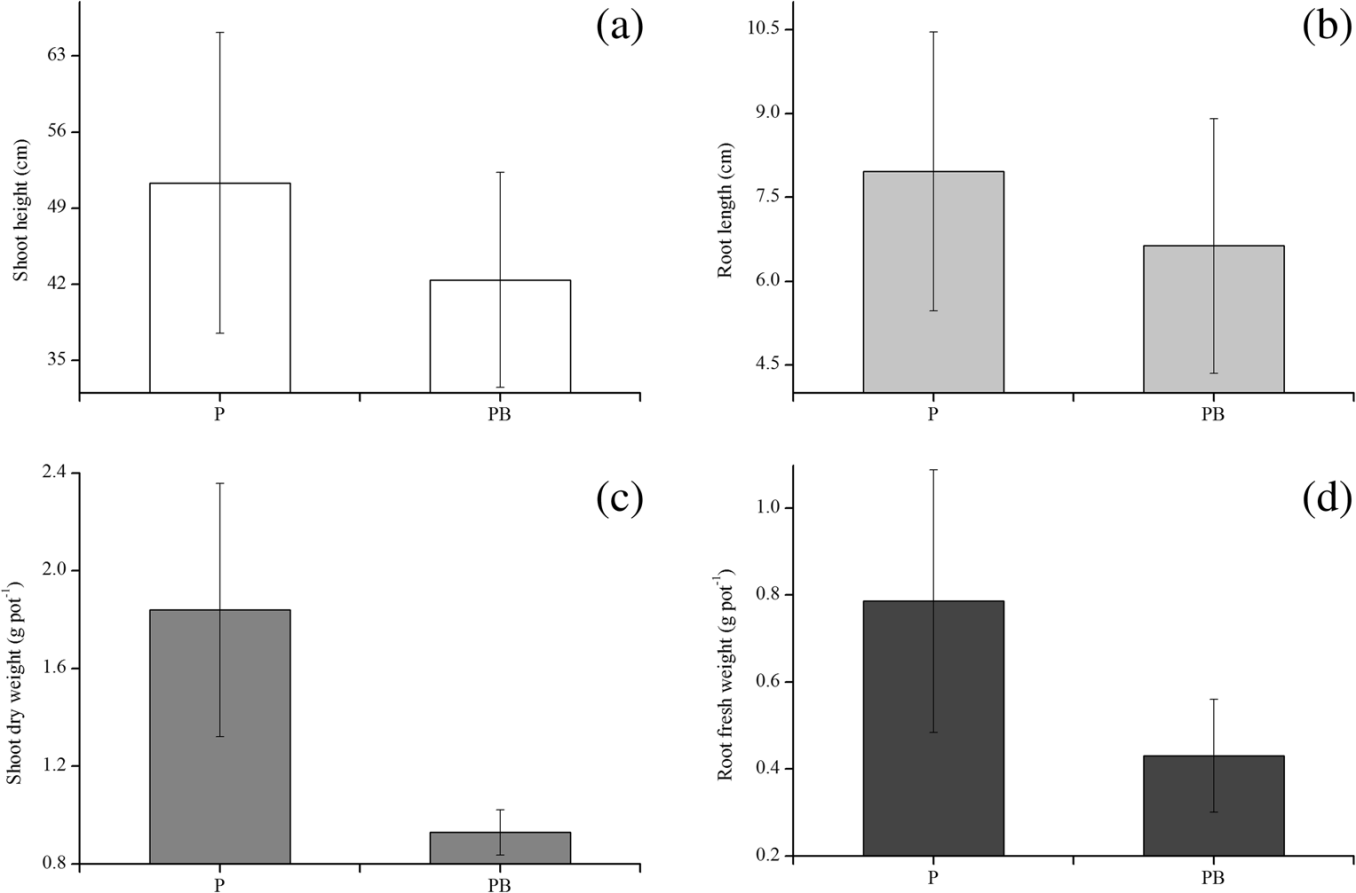
Growth parameters of ryegrass, including shoot height, root length, shoot dry weight, and root dry weight, with (treatment PB) or without (treatment P) the amendment of biochar. *Error bars* represent the standard deviation

Four parameters (e.g., shoot height, shoot dry weight, root length, and root fresh weight) were measured to assess the growth of ryegrass (Fig. 2). The amendment of biochar showed a negative effect on the growth of ryegrass (Fig. 2). There were significant differences between the shoot height, root length, and shoot dry weight of ryegrass in treatment PB and those in treatment P (*p* < 0.05). After 90 days, the shoot height, root length, and shoot dry weight in treatment PB were 17.38, 16.74, and 49.41 % lower than those in treatment P, respectively. In contrast, there was no significant difference in the root fresh weight of ryegrass between treatments PB and P.

**Fig. 3:**
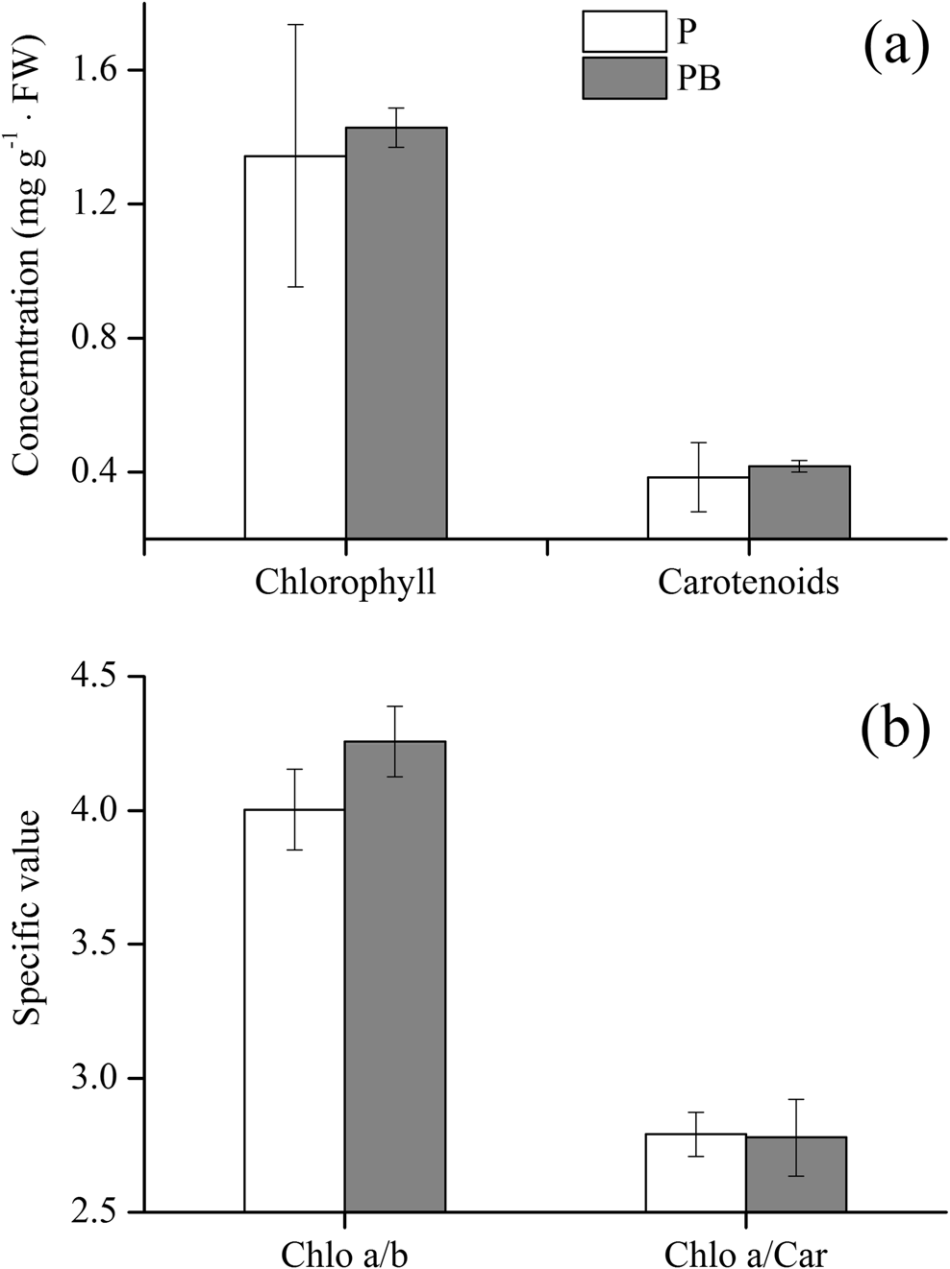
Photosynthetic pigments including total chlorophyll, total carotenoids, chlorophyll a/b ratio, and chlorophyll a/carotenoid ratio of ryegrass with (treatment PB) or without (treatment P) the amendment of biochar. *Error bars* represent the standard deviation

Pigment content is one of the indicators of the cellular metabolic state. As shown in Fig. 3, all pigment parameters including chlorophyll, carotenoids, chlorophyll a/chlorophyll b, and chlorophyll a/carotenoids showed no significant differences (*p* > 0.05) between treatments P and PB.

### Component changes in the process of phytoremediation

The contents of petroleum hydrocarbon components decreased with time. The removal rate of total n-alkanes in treatment P (45.83 %) was significantly higher (*p* < 0.01) than that in other treatments, and no significant differences were observed among the rest of the treatments (Fig. 1). For treatments C, B, and PB, the removal rate was 5.57, 8.53, and 7.93 % lower than that in treatment P, respectively. Furthermore, there was a component change in n-alkane before and after incubation. The main n-alkane content after 90 days of incubation was C_14_ to C_40_. Moreover, the major removal of n-alkanes in treatment P was C_39_ (Table 2).

**Table 2:** Concentration of various components of *n*-alkanes (μg/kg·dry soil) before incubation (day 0) and after 90 days of incubation in different treatments (C, P, B, PB)

*n*-Alkanes	Day 0 (μg/kg dry soil)	C (μg/kg dry soil)	P (μg/kg dry soil)	B (μg/kg dry soil)	PB (μg/kg dry soil)
C8	25 ± 3	54 ± 8	43 ± 1	53 ± 1	56 ± 4
C9	17 ± 3	16 ± 5	11 ± 2	13 ± 0	16 ± 3
C10	4 ± 1	6 ± 0	6 ± 0	7 ± 0	7 ± 0
C11	14 ± 3	26 ± 1	31 ± 14	27 ± 2	26 ± 1
C12	14 ± 4	19 ± 1	17 ± 2	18 ± 2	17 ± 1
C13	39 ± 17	27 ± 5	23 ± 3	32 ± 5	23 ± 0
C14	309 ± 107	145 ± 61	153 ± 99	301 ± 205	133 ± 76
C15	787 ± 172	312 ± 148	300 ± 111	428 ± 225	313 ± 125
C16	1923 ± 347	1140 ± 326	1095 ± 229	1370 ± 498	1272 ± 293
C17	2881 ± 355	1725 ± 390	1581 ± 228	1810 ± 459	1889 ± 148
C18	3121 ± 352	2478 ± 293	2172 ± 205	2505 ± 225	2618 ± 155
C19	3613 ± 340	2836 ± 258	2515 ± 114	2833 ± 192	2924 ± 107
C20	5749 ± 598	4711 ± 183	4242 ± 88	4774 ± 38	4914 ± 122
C21	6752 ± 648	5658 ± 68	5068 ± 43	5671 ± 17	5842 ± 123
C22	7662 ± 746	6655 ± 209	6029 ± 36	6687 ± 101	6922 ± 235
C23	8885 ± 818	7781 ± 207	7157 ± 66	7744 ± 89	8086 ± 164
C24	9661 ± 994	8380 ± 272	7714 ± 105	8204 ± 94	8642 ± 155
C25	11,058 ± 1315	9251 ± 300	8591 ± 130	8991 ± 192	9448 ± 77
C26	11,866 ± 1192	9353 ± 309	9198 ± 86	9068 ± 179	9672 ± 311
C27	10,598 ± 1166	7731 ± 310	7256 ± 69	7529 ± 177	7835 ± 51
C28	9092 ± 1119	6073 ± 257	5715 ± 83	5819 ± 82	6138 ± 22
C29	9436 ± 1412	5853 ± 283	5534 ± 103	5719 ± 180	5914 ± 37
C30	10,906 ± 1855	6237 ± 393	5975 ± 390	6269 ± 586	6754 ± 16
C31	11,111 ± 1473	5892 ± 220	5620 ± 194	5890 ± 184	5985 ± 48
C32	8257 ± 1319	3802 ± 296	3737 ± 131	4065 ± 222	3992 ± 27
C33	8879 ± 1530	3809 ± 241	3634 ± 125	3996 ± 138	4007 ± 117
C34	7995 ± 1573	3015 ± 203	2795 ± 154	3287 ± 123	3236 ± 74
C35	8132 ± 1489	3072 ± 249	2868 ± 136	3380 ± 135	3237 ± 22
C36	7246 ± 1430	2144 ± 187	1940 ± 133	2486 ± 119	2320 ± 69
C37	12,453 ± 2504	3662 ± 375	3227 ± 183	4305 ± 204	3997 ± 149
C38	7916 ± 1194	2399 ± 248	2097 ± 94	2770 ± 165	2552 ± 113
C39	8537 ± 1280	2378 ± 163	2098 ± 66	2609 ± 124	2008 ± 790
C40	10,154 ± 568	5893 ± 1093	2667 ± 971	10,241 ± 454	6867 ± 517
Sum	205,100 ± 27,927	122,533 ± 6233	111,109 ± 463	128,901 ± 3785	127,662 ± 1339

**Fig. 4:**
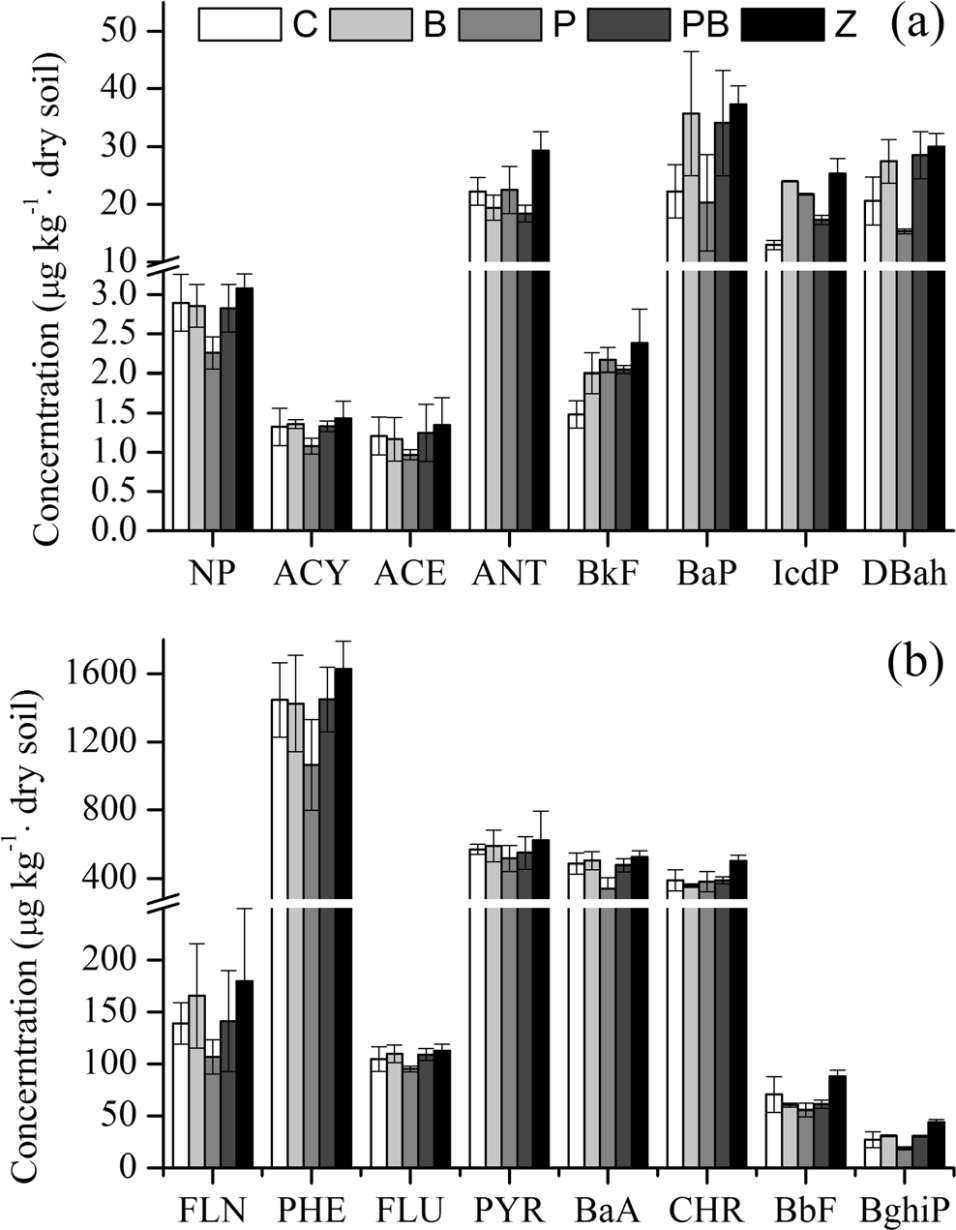
Components of PAHs at day 0 (labeled as *Z*) and after 90 days of incubation in four different treatments (i.e., C, B, P, PB). *NP*: naphthalene; *ACY* acenaphthylene; *ACE* acenaphthylene; *FLN* fluorene; *PHE* phenanthrene; *ANT* anthracene; *FLU* fluoranthene; *PYR* pyrene; *BaA* benzo(a)anthracene; *CHR* chrysene; *BbF* benzo(b)fluoranthene; *BkF* benzo(k)fluoranthene; *BaP* benzo(a)pyrene; *IcdP* indeno(1,2,3-cd)pyrene; *DBah* dibenzo(a,h)anthracene; *BghiP* benzo(ghi)perylene. *Error bars* represent the standard deviation from triplicate measurements

The highest removal rate of total PAHs was also observed in treatment P (30.34 %). There were no significant differences among the rest of the treatments. For treatments C, B, and PB, the removal rate was 16.97, 17.14, and 16.04 % lower than that in treatment P, respectively. The removal rate of total n-alkanes was greater than that of the total PAHs after a 90-day incubation period (Fig. 1). Specifically, the total PAH content in all of the soils was 36–53 times lower than that of the total n-alkanes in each treatment, indicating that the removal of n-alkanes made a major contribution to the removal of TPHs. Furthermore, there was no clear component change in PAHs compared to n-alkanes. As shown in Fig. 4, four components (i.e., phenanthrene (PHE), pyrene (PYR), benz(a)anthracene (BaA), and chrysene (CHR)) accounted for 85.52–87.17 % of the total PAHs in all treatments before and after incubation. The majority of the removal was also attributed to these four components (i.e., 34.58 % for PHE, 16.93 % for PYR, 34.78 % for BaA, and 23.89 % for CHR) in treatment P.

The content of total n-alkanes and total PAHs in biochar was 47,100 and 3484 μg/kg, accounting for 0.23 and 0.91 % of those in the control at the start of the experiment, respectively. The main n-alkanes of biochar in soil/control soil were from C_8_ to C_13_, which accounted for 1.52–7.03 %. The main PAHs of biochar in soil/control soil were benzo(k)fluoranthene (BkF), benzo(a)pyrene (BaP), indeno(1,2,3-cd)pyrene (IcdP), and benzo(ghi)perylene (BghiP), which accounted for 37.69, 12.15, 9.31, and 4.82 %, respectively.

### Effects on total microbial count

The concentration of microorganisms in soil contaminated with petroleum hydrocarbons was assessed and enumerated before and after remediation. A significant increase of microorganisms (from 2.91 × 10^6^ to 1–4 × 10^7^ cells/g dry soil) was observed in all treatments, suggesting growth of microorganisms on TPHs. In addition, significant differences (*p* < 0.01) in microbial biomass after incubation for 90 days were observed among the four treatments (Fig. 1). The number of microorganisms in treatment P (3.93 × 10^7^ cells/g dry soil) was significantly higher (*p* < 0.01) than in the other three treatments. Specifically, the number of microorganisms in treatment P was 2.09, 2.05, and 1.54 times higher than that of treatments C, B, and PB, respectively. The lowest microbial concentration was observed in treatment C (1.27 × 10^7^ cells/g dry soil) where no amendment was added. Statistically, there were no significant differences (*p* > 0.05) among the microbial concentrations in treatments C, B, and PB after the 90-day incubation.

## Discussion

The current study reports the efficiency of three petroleum-contaminated soil remediation strategies. The control treatment achieved 30 % of TPH removal after 90 days of incubation, which is comparable to previous reports (Cai et al. 2010; Zhang et al. 2010). Fertilizer was applied at the beginning of the experiment, which could significantly enhance the microbial degradation of TPHs. It was reported that fertilizer application could promote the quantity of microorganism and bioremediation rates of environments polluted with petroleum hydrocarbons (Nikolopoulou et al. 2009; Mrozik et al. 2003). For example, it was observed that bacterial populations were stimulated by controlled release fertilizer application during phytoremediation of TPHs (Cartmill et al. 2014). In addition to microbial degradation, volatilization, eluviation, and photolysis were reported to play an important role in the removal of petroleum hydrocarbons (Zhang et al. 2010), which can also promote removal of TPHs in the control treatment.

The addition of biochar (treatment B) did not increase the effectiveness of TPH removal (Fig. 1), which is consistent with a previous study (Carcia-Delgado et al. 2015). Biochar contains many pores that may affect its function in the soil, e.g., smaller pores may influence molecule adsorption and transport, while larger pores are significant for water-holding capacity and aeration of soil. In essence, biochar contains a greater surface area because its pores have a far larger concentration than soil (Quilliam et al. 2013a). The porous physical properties of biochar provide an important habitat niche for microorganisms, which may be the most common explanation for why biochar can increase the abundance and activity of soil microorganisms (Pietikäinen et al. 2000). However, its contribution is small (Quilliam et al. 2013a). Studies have shown that biochar barely affects the composition of the soil microbial community (Steinbeiss et al. 2009). Our study has shown that after burial in the soil for 3 months, despite the significant reduction in the number of microorganisms compared to the plant treatment, biochar had no significant impact on other treatments. This was less likely to be due to surface area and pore volume in our study. Biochar could also affect soil properties, e.g., significantly increased soil pH and soil C, increased sub-nanopore surface area, reduced soil bulk density, and so on (Mukherjee et al. 2014). However, relative to the soil, the contribution of biochar is very low in the total soil pore space and the total soil surface area. Furthermore, a large proportion of the biochar pores were less than 1 μm in diameter, which is effectively uninhabitable for most microbes. Biochar has a lower available C status and differs in the structure of the microbial community relative to the surrounding soil. At least in the short term, biochar cannot provide a meaningful habitat for microbes (Quilliam et al. 2013a). One reason may be that biochar often introduces high concentrations of mineral salts and PAHs, which can have a negative impact on the microbial colony (Boonchan et al. 2000).

The results obtained in this study offer clear evidence that phytoremediation with ryegrass was more efficient in the removal of TPHs than the other treatments used (Fig. 1). It was reported that ryegrass could be applied to the phytoremediation of hydrocarbon-polluted soil (Arslan et al. 2014) and herbicide-contaminated solutions (Mimmo et al. 2015). The fact that majority of the removal of n-alkanes happened to C_39_ (Table 2) indicates that ryegrass has the potential to be applied in the phytoremediation of long-chain alkanes. Furthermore, four major components of PAHs accounted for about 80 % of the total removal of PAHs. The results are consistent with previous report where PHE, PYR, and CHR contributed to 75 % of the PAH removal (Wang et al. 2012).

Plants have been shown to play an important role in the process of remediation (Liu et al. 2014; Rodríguez-Vila et al. 2014). Plants may add an increased number of microorganisms, improve soil physicochemical properties, and increase the humification and adsorption of pollutants in the rhizosphere to cause the removal of petroleum hydrocarbons (Al-Mansoory et al. 2015). In the current study, the ryegrass showed a promoting effect on the removal of petroleum hydrocarbons in soil. This may be due to the joint actions of plants and microorganisms (Khan et al. 2013; Zhang et al. 2010). The microbial number also reached the highest in treatment P (Fig. 1). It was reported that plants and their associated bacteria interact with each other during phytoremediation (Khan et al. 2013). Plant growth can provide nutrition and promote growth of rhizosphere microorganisms. In return, microorganisms can increase the plant nutrient supply, and adding plant resistance to poisons or reducing poisonous bioavailability in the rhizosphere may help plant growth. For example, some plant-associated bacteria can produce biosurfactants that can enhance the bioavailability of hydrocarbons and may be useful for phytoremediation applications (Pacwa-Płociniczak et al. 2011). These microorganisms can secrete plant hormones and promote the absorption of nutrients in the soil, which not only directly stimulates plant growth but also improves the adaptation of plants to drought, salinity, and toxicity of metals and organic pollutants (Das and Tiwary 2014). Taken together, the synergistic effects between plants and their associated microorganisms is a feasible “clean up” technology in the removal of hydrocarbon pollutants for the remediation of contaminated soils (Gaskin and Bentham 2010).

The results presented here showed that the amendment of biochar played a negative role in the phytoremediation of petroleum-contaminated soil (Fig. 1) as well as the growth of ryegrass (Figs. 2 and 3). It is known that biochar contains petroleum hydrocarbons which may have toxicity to plants and microorganisms (Oleszczuk et al. 2013; Quilliam et al. 2013b). During a slow and long pyrolysis, PAHs are more easily lost to the atmosphere, whereas they are more readily concentrated in the biochar surface during a fast pyrolysis (Hale et al. 2012). However, compared to petroleum hydrocarbons applied to the soil in the current study, the alkanes (47 mg/kg) and PAHs (3.5 mg/kg) contained in the biochar can be neglected. It is therefore possible that other properties of the biochar affected the removal of petroleum hydrocarbons. Biochar in the soil can strongly adsorb soil nutrients and organic matter, which can jam the pores and reduce the available pore volume and surface area of biochar (Joseph et al. 2010). In addition, biochar may reduce soil nutrient leaching (e.g., nitrate, ammonium, phosphate, and microelement) (Beesley and Marmiroli 2011; Gomez-Eyles et al. 2011; Guo et al. 2014; Ippolito et al. 2012; Schmidt et al. 2014; Yao et al. 2012) and can impact C and nutrient bioavailability in the charosphere (Quilliam et al. 2013a), which limits the use of nutrients in plants and microorganisms.

It was reported that biochar did not have a significantly positive effect on plant yield (Evangelou et al. 2014; Lucchini et al. 2014; Mia et al. 2014; Schmidt et al. 2014). Biochar can increase the sorption of organic pollutants (Chen and Yuan 2011; Ippolito et al. 2012; Oleszczuk et al. 2012; Quilliam et al. 2013b; Tang et al. 2013). In particular, PAHs adsorbed by biochar per unit mass can be 10–1000 times more than other types of organic C in soils (Accardi-Dey and Gschwend 2003; Rhodes et al. 2010). A large number of studies have shown that biochar can also reduce bioavailability of organic pollutants (Ippolito et al. 2012; Quilliam et al. 2013b) through adsorbing nutrients (Joseph et al. 2010) and producing toxicity to microorganisms (Oleszczuk et al. 2013; Quilliam et al. 2013b). In general, biochar may make the soil a nutrient poor and potentially toxic environment for plants and microorganisms to colonize.

## Conclusions

This work demonstrated that bioremediation strategies for petroleum-contaminated soil (1 %) with phytoremediation using ryegrass were more efficient than non-amended soil or biochar application. The phytoremediation with ryegrass achieved effective removal of petroleum hydrocarbons (55.13 % for TPHs, 30.34 % for PAHs, and 45.83 % for n-alkanes) over a 90-day incubation period. It was clearly shown that ryegrass could promote the growth of microorganisms in the contaminated soils with 10,000 mg/kg of TPHs. Although the amendment of biochar did not cause significant negative influences on soil microflora, it suppressed the growth and development of ryegrass. In general, ryegrass can effectively promote the removal of TPHs for petroleum-contaminated soil, and the removal efficiency was restrained under the effects of biochar. Our results suggest that the amendment of biochar is not suitable for phytoremediation of petroleum-contaminated soil.
